# Farber disease: understanding a fatal childhood disorder and dissecting ceramide biology

**DOI:** 10.1002/emmm.201302781

**Published:** 2013-05-13

**Authors:** Mark S Sands

**Affiliations:** Departments of Internal Medicine and Genetics, Washington University School of MedicineSt. Louis, MO, USA

**Keywords:** acid ceramidase, ceramide, Farber disease, lysosomal storage disease

See related article in EMBO Molecular Medicine http://dx.doi.org/10.1002/emmm.201202301

Farber disease (Lipogranulomatosis) is a rare, invariably fatal, inherited metabolic disorder first described by Sidney Farber in 1957 (Farber et al, 1957). Farber disease is inherited in an autosomal recessive fashion and is caused by mutations in the lysosomal acid ceramidase (*ASAH1*) gene. Therefore, Farber disease is classified as one of the >50 distinct lysosomal storage disorders (LSDs). Acid ceramidase (ACDase) is a soluble lysosomal enzyme responsible for the degradation of ceramide. Low levels (≤10% normal) of ACDase activity result in the progressive accumulation of ceramide in most tissues.

Affected children initially present with joint stiffness and deformation, prominent subcutaneous nodules and progressive hoarseness due to laryngeal involvement. As the disease progresses affected children can also develop cardiac, pulmonary and central nervous system defects. Like most LSDs, there is a spectrum of severities associated with Farber disease. The most severe neonatal form results in death within the first few days of life. Children with the most common, ‘classical’ form of Farber disease present symptoms by 3–6 months of age and die at approximately 2 years of age. The intermediate/mild form has no neurologic component and affected patients typically live to 5–7 years of age. However, some mild Farber patients live into their teens or early adulthood. The pathogenesis of Farber disease is largely unknown. In addition, there is no apparent genotype/phenotype correlation and little or no correlation between ACDase levels and disease severity. This lack of understanding is due in part to the small number of patients evaluated and the imprecision associated with the ACDase assays.

» This represents a truly significant advancement in the study of this disorder. «

An authentic small animal model of Farber disease would greatly facilitate research into disease mechanisms and aid in the development of effective therapies. Unfortunately, a complete ‘knock-out’ model of Farber disease resulted in early embryonic lethality (Li et al, 2002) and a reduction of ACDase activity in the ovaries of a conditional ‘knock-out’ resulted in oocyte apoptosis (Eliyahu et al, 2012). These data highlight the difficulty in generating mouse models of certain LSDs, in particular the lipid storage disorders. These data also suggest that normal ceramide levels and metabolism are critical for early embryonic development.

Significant progress has been made towards effective therapies for LSDs through the use of recombinant enzyme replacement, bone marrow transplantation (BMT), small molecule drugs and gene therapy. It is likely that Farber disease would be responsive to similar approaches. However, the lack of an authentic animal model of Farber disease has hindered this research. Thus, there is no effective therapy currently available for Farber disease.

In this issue of EMBO Molecular Medicine, Alayoubi et al (2013) describe the development of a ‘knock-in’ mouse model of Farber disease. This represents a truly significant advancement in the study of this disorder. Since no complete *ASAH1* deletions have been identified to date in Farber patients, the authors inserted a homoallelic missense mutation (P362R) discovered in a patient with a ‘classical’ Farber disease presentation. This particular mutation is located within the most highly conserved amino acid region of the mouse and human *ASAH1* genes. This mutation results in normal levels of protein but <10% normal ACDase activity in an *in vitro* expression assay (Li et al, 1999). Alayoubi et al (2013) performed a biochemical, histological, haematological and clinical evaluation of the *Asah1*^*P361R/P361R*^ mouse in order to determine if this model mimics the human disease. Briefly, the mouse has low levels of ACDase activity, ceramide accumulation in all tissues assayed (including the brain), histiocytic infiltrations, decreased body weight, shortened life span (median ≍65 days), impaired ovary development, altered myeloid parameters, shortened epiphyseal plates and elevated MCP-1 levels in liver, spleen, brain and thymus. Finally, the authors demonstrate that the *Asah1*^*P361R/P361R*^ mice have profound hydrocephalus as measured by magnetic resonance imaging. Based on this initial yet extensive characterization, it is clear that the ACDase-deficient mouse accurately models most of the characteristics of the human disease. It will be of great interest to more thoroughly investigate the effects of lysosomal ceramide accumulation on the animal's metabolic state, skeletal development, immune system, and CNS function.

Not only did Alayoubi et al (2013) create and characterize a mouse model of Farber disease, in the same report they performed an experiment to determine the efficacy of systemic gene therapy for this disease. The authors injected a lentiviral vector expressing human ACDase intravenously into neonatal *Asah1*^*P361R/P361R*^ mice. This approach was chosen for two reasons: (i) Farber disease is progressive and early treatment will likely be more efficacious since it can prevent rather than reverse pre-existing disease, and (ii) ACDase is a soluble lysosomal enzyme that can be secreted from the genetically modified cells and taken up by non-transduced cells through a receptor-mediated mechanism. This has the potential to mediate widespread correction of the disease (Sands and Davidson, 2006). The lentiviral-treated mice had significantly increased body weight and life span, improved haematologic parameters, and nearly normal ceramide levels in the liver and spleen. Unfortunately, there was no decrease in ceramide accumulation in the brain. Although the therapeutic response was only partial, this is an important proof-of-principle experiment demonstrating the feasibility of this approach and could not have been performed without the *Asah1*^*P361R/P361R*^ mouse. The creation of an accurate small animal model of Farber disease will allow the investigators to identify new disease mechanisms that can be targeted either alone or in combination, thus increasing efficacy (Hawkins-Salsbury et al, 2011).

» It will be of great interest to more thoroughly investigate the effects of lysosomal ceramide accumulation on the animal's metabolic state, skeletal development, immune system and CNS function. «

**Figure 1:**
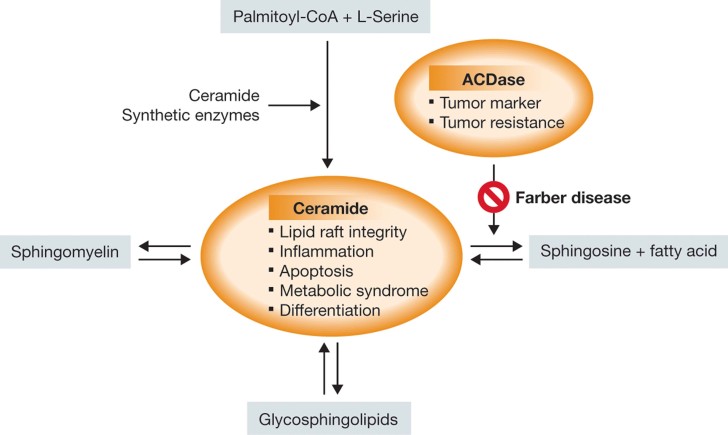
Acid ceramidase and ceramide actions Ceramide is a lipid that is sequentially synthesized through several reactions starting with palmitoyl-CoA and l-serine. Ceramide is central to the further synthesis of several key membrane lipids such as sphingomyelin and the glycospohingolipids, galactosylceramide and glucosylceramide. Ceramide has also been shown to be directly involved in a number of biological processes, including lipid raft integrity, inflammation, apoptosis, metabolic syndrome and differentiation, just to name a few. Acid ceramidase (ACDase) is a soluble lysosomal enzyme responsible for the breakdown of ceramide to sphingosine and fatty acid. A deficiency in ACDase activity leads to the accumulation of ceramide in many tissues, the hallmark of Farber disease. Acid ceramidase has also been shown to be involved in the resistance of certain tumor cells to chemotherapeutic agents and has been identified as a tumor marker in other malignancies.

The implications of the *Asah1*^*P361R/P361R*^ mouse for understanding the pathogenesis of, and developing therapies for Farber disease are obvious. However, this model also represents a powerful tool to aid in the understanding of ceramide biology. There is a growing body of literature demonstrating the importance of ACDase, ceramide, and related lipids in a number of biological processes (Fig 1). ACDase is one of the key enzymes regulating ceramide and sphingolipid metabolism and has been shown to be involved in tumorigenesis (Park & Schuchman, 2006). Over expression of ACDase has been identified as a marker of certain tumours and can inhibit tumour cell apoptosis. Inhibition of ACDase activity is being actively investigated as a possible therapy for certain tumours. The *Asah1*^*P361R/P361R*^ mouse will help elucidate the role of ACDase, ceramide and other sphingolipids in tumorigenesis and allow therapeutic approaches targeted at ACDase to be tested *in vivo*.

There are a relatively large number of functions ascribed directly to ceramide and it also serves as the core molecule for a number of sphingolipids, including sphingomyelin and the glycosphingolipids (Fig 1). Ceramide has been shown to be a pro-apoptotic molecule. This is consistent with the oocyte apoptosis observed in the conditional *Asah1* ‘knock-out’ described above (Eliyahu et al, 2012). Ceramide can also have direct effects on inflammation, immune function, cardiac function, atherosclerosis, metabolic abnormalities and differentiation, just to name a few (Bieberich, 2012). Although *in vitro* data suggest that lysosomal ceramide accumulation in cultured Farber cells does not affect normal ceramide signalling pathways, it seems unlikely that the ceramide that accumulates in diseased cells or is released from dead or dying cells will be completely benign *in vivo*. Ceramide, sphingomyelin and the glycosphingolipids, galactosylceramide and glucosylceramide, are integral membrane components that are concentrated in lipid rafts and are believed to be important for both proper raft and non-raft architecture. Sphingomyelin and galactosylceramide are also two of the major components of myelin sheaths surrounding axons. Galactosylceramide, in particular, makes up >20% of the total mass of myelin lipids. Perturbation of ceramide levels as a result of altered catabolism by ACDase could affect the levels of the glycosphingolipids and sphingomyelin, and, secondarily membrane integrity, the proper functioning of raft- or membrane-associated proteins, and axonal health. The ACDase-deficient mouse represents an important addition to a growing number of mouse models that have genetic defects affecting sphingolipid metabolism (Sabourdy et al, 2008). These models, alone and in combination will facilitate both the study of the physiological consequences of lysosomal ceramide accumulation and the dissection of these complex lipid signalling pathways *in vivo*.

In conclusion, Alayoubi et al (2013) thoroughly and convincingly demonstrate that the mouse model of ACDase deficiency is an accurate phenocopy of Farber disease and systemic gene therapy may be part of an effective therapeutic strategy. This animal model will also provide a new and important tool in the quest to more fully understand the complex actions of ceramide and sphingolipids.

» This animal model will also provide a new and important tool in the quest to more fully understand the complex actions of ceramide and sphingolipids. «
